# Birth preparedness and complication readiness among husbands and its association with skilled birth attendance in southern Ethiopia

**DOI:** 10.1186/s12884-022-05147-3

**Published:** 2022-11-19

**Authors:** Yordanos Gizachew Yeshitila, Peter Memah

**Affiliations:** 1 School of Nursing, College of Medicine and Health Science, Arba Minch University, Arba, Minch Ethiopia; 2 Division of Epidemiology and Prevention, Institute of Human Virology, University of Maryland School of Medicine, Baltimore, MD USA; 3 Deakin Health Economics, Institute for Health Transformation, Deakin University, Melbourne, Victoria Australia

**Keywords:** Birth preparedness and complication readiness, Husband involvement, Skilled birth attendant, Southern Ethiopia

## Abstract

**Introduction:**

Birth preparedness and Complication Readiness (BPCR) promotes prompt use of skilled delivery and readiness for any complications to reduce delays in the provision of emergency care. Husband’s involvement in preparation for childbirth is one way to mitigate life-threatening delays in providing care during childbirth. The current study assessed the association of the husband’s involvement in birth preparedness and complication readiness with the use of skilled birth attendants.

**Method:**

A community-based cross-sectional study was conducted among 1,432 husbands. Study participants were selected from Arbaminch university health and demographic surveillance sites. Data were collected electronically using the Open Data Kit. The data were exported to STATA version 16 for analysis. Descriptive statistics were computed to describe the sociodemographic and reproductive variables of the study. The associations between birth preparedness and complication readiness practice and birth in the presence of a skilled birth attendant were assessed using multiple logistic regressions after adjusting for known confounders. Explanatory variables on bivariate logistic regression analysis were entered into multivariable logistic regression analysis, and a p-value of less than 0.05 was used to ascertain statistical significance.

**Results:**

Mean age of respondents was 33.7 (SD ± 6.2) years. Of all the respondents, 140 (10.3%) had made at least three preparations according to birth preparedness and complication readiness. After controlling for confounders through multivariable logistic regression, giving birth in the presence of a skilled birth attendant consistently increased with husbands involved in birth preparedness and complication readiness (AOR = 4.1, 95% CI: 2.5–6.9). Husbands whose wives had complications during previous pregnancy were 33% less likely to have skilled birth attendants (AOR = 0.67, 95% CI: 0.49–0.917). Moreover, husbands whose houses were near the health facilities were more likely to have skilled birth attendants for their wives (AOR = 3.93, 95% CI: 2.57–6.02).

**Conclusion:**

Husband’s involvement in birth preparedness and complication readiness is strongly associated with using skilled birth attendants in Ethiopia. It is imperative that when designing husband’s involvement programs targeting birth preparedness and complication readiness, programs and strategies should focus on enhancing activities that are inclusive of husbands in birth preparedness and complication education.

**Supplementary Information:**

The online version contains supplementary material available at 10.1186/s12884-022-05147-3.

## Introduction

Birth preparedness and Complication Readiness (BPCR) is conceptualized using six main variables, including a preparing birth kit, identifying a skilled attendant, saving money, knowing where to go in cases of emergency, contacting a blood donor in advance, and preparing transportation in advance [1]. This promotes prompt use of skilled delivery and readiness for any complications to reduce delays in the provision of emergency care. A systematic review indicated that BPCR interventions reduce maternal and neonatal health risks, especially in low-income countries [2]. BPCR programs primarily focus on emergency obstetric care and skilled delivery during childbirth. Yet, other yet other contextual societal factors such as male involvement play a crucial role in ensuring that the two components mentioned above are successfully achieved.

The concept of male involvement in maternal health, which includes the shared responsibility of men to engage in responsible prevention of high-risk pregnancies actively, maternal and child health, family planning, parenthood, and prevention of sexually transmitted diseases, has been recognized in recent years [3]. One of the critical reasons why pregnant women do not seek maternity services is the absence of support from their husbands [4], who are key decision-makers, especially in the patriarchal societies of sub-Saharan Africa.

Efforts to understand and improve the extent of men’s responsibilities for their health and that of their partners are growing [3]. Findings from the World Health Organization (WHO) meeting [3] on male involvement, indicated that in terms of safe motherhood: (1) Men should not be seen as passive bystanders and barriers, instead they have a unique role in promoting safe motherhood, (2) men should be informed of the social, economic and cultural complexities of safe motherhood as they are similarly affected [5], (3) when their spouses, siblings or mothers die, they are often affected and need to understand the factors that contribute, and (4) little support is given to men to encourage their involvement in care yet some men are receptive and eager to participate in safe motherhood. Despite the aspects highlighted, it is evident that male partner involvement in maternal health has significantly contributed to improved utilization of institutional delivery [6]. Additionally, other benefits of involving men in maternity care have increased access to antenatal visits hence increased the likelihood of access to skilled birth attendants, family planning, and addressing gender-based barriers to access to maternal health [7–9].

In 2015, the WHO recommended the use of, BPCR stating that “programs that involve BPCR interventions are recommended to increase the use of skilled care at birth and to increase the timely use of facility care for obstetric and newborn complications” (1), and the WHO included it as an integral component of antenatal care (2). A study conducted in southern Ethiopia on husbands’ involvement during antenatal care was proven to be associated with skilled birth attendance (3). Given that majority of maternal deaths and obstetric complications occur during childbirth, skilled birth attendance is the most important intervention (4).

A systematic review of interventions conducted in low and middle-income countries highlighted that engaging men in maternal and child health services were associated with improved antenatal care attendance, skilled birth attendance, facility birth, postpartum care, birth and complications preparedness, and maternal nutrition [4, 5]. Similarly, an interventional study reported a positive association between male involvement in maternal health and positive maternal health behaviors, especially related to the utilization of services and preparation for facility delivery [6, 10].

A higher proportion of male involvement was reported among primiparous women living in developed countries and planned pregnancies [9]. However, sadly in developing countries, male involvement in BPCR has been slow. Very few studies have explored the role of men in birth preparedness and complication readiness. A study on male involvement in maternity care in Tanzania indicated that only 20% of 996 were involved in maternity care [5]. Another study indicated that a small proportion of men had prepared for childbirth and complication readiness, with half having bought kits, 47.2% saved funds, 10.2% identified transport, and 0.8% identified skilled attendants [1, 11].

Several factors have been explored to establish the extent of husbands’ involvement in maternity care and BPCR. A study in Tanzania indicated that the number of children, limited access to information, and limited spousal communication decreased male involvement [5]. Similarly, improved knowledge of obstetric danger signs helps husbands support their partners in accessing Antenatal Care (ANC) and delivery care [1]. Another study indicated that BPCR knowledge among men is positively influenced by having heard of birth preparedness, age at marriage, and educational status [12, 13]. Additionally, the outcomes that are often focused on are not substantive in terms of how men and women relate but ideally focus on specific support instances like saving money for transport in case of birth complications [7]. A systematic review on the impact of male involvement around the time of childbirth on Maternal, Newborn, and Child Health (MNCH) outcomes revealed gaps in male involvement strategies and differences in social and cultural contexts [7].

In Ethiopia, to reduce maternal mortality to 199 maternal deaths per 100,000 live births and neonatal mortality to 10 per 1,000 live births by 2020, a series of high-impact interventions have been performed, including prenatal care, skilled birth attendant, and postnatal care [7]. However, according to recent national health and demographic survey, less than half of births have a skilled birth attendants [8].

In Ethiopia, BPCR planning have been incorporated as essential component in delivering focused antenatal care and have been prioritized as a key strategy for promoting skilled birth attendants [15]. Despite few studies conducted to assess husband involvement in birth preparedness and complication in Ethiopia [16, 17, 18, 19], there is no evidence on how husband involvement in birth preparedness and complication contributes to the use of skilled birth attendants. Therefore, this paper highlights husband involvement in BPCR and its association with skilled birth attendance in southern Ethiopia.

## Materials and methods

### Study setting and design

A community-based cross-sectional study was conducted at Arba Minch Demographic and Health Surveillance Site (HDSS) from October 1, 2020, to December 30, 2020. The surveillance site is found in Arba Minch zuria district with the administrative center of Arba Minch town, which is located 505 km southwest of Addis Ababa, the capital city of Ethiopia, and 275 km southwest of Hawassa, the capital city of Southern Nation Nationalities and Peoples Region (SNNPR). The district has a total of 29 kebeles (the smallest administrative unit in the current Ethiopian government structure under the district); two of which are semi-urban, and the remaining 27 are rural. The Arba Minch HDSS site operates in nine, district kebeles randomly selected after stratifying the districts into highland and lowland climatic zones. The surveillance site tracks demographic changes like death, birth, migration, marital status change, nutrition, reproductive health, morbidity/health-seeking behavior, and health care utilization by permanently recruited data collectors who use the Open Data Kit (ODK) mobile phone-based application for data collection and report monthly to the center located at Arba Minch University [20, 21, 22].

### Population

The source population was all men in marital union with a woman (legal, religious, or traditional) having children less than one year of age and permanent residents of the Arba Minch zuria district. All men in a marital relationship having children less than one year of age and permanent residents of Arba Minch zuria district and selected during the study period were the study population.

### Sample size and sampling procedure

The sample size for this study was initially calculated using the stat calc menu of Epi-info software version 7 [23] using the assumptions for the single population proportion formula based on the prevalence of 34.8%, 95% confidence level of Za/2 = 1.96, 5% of absolute precision, and 10% nonresponse rate [24], the calculated sample size was 349. Additionally, sample sizes were calculated using factors associated with husband involvement in skilled birth attendants, such as escorted wives to ANC in the previous pregnancy, knowledge of at least one danger sign during pregnancy, and knowledge of at least one danger sign during postpartum. The largest sample size(n = 1302) was obtained by considering 12.9% skilled birth attendants among husbands who escorted wives to ANC in the previous pregnancy, AOR of 1.7 [12], 80% power, 95% confidence level, 5% degree of precision, and the ratio of unexposed to exposed is equal to 1. After adding a non-response rate of 10%, the sample size calculated using two- a population proportion (1432) was used for this study.

All nine kebeles under Arba Minch HDSS were included. A total of 1652 husbands in a marital relationship with children less than one year of age and permanent residents that fulfill the inclusion criteria were selected. A sampling frame (secondary data was used from the HDSS site database) of these 1652 husbands was used to determine the sample for this study. The sample size for each kebeles was proportionally allocated based on the information obtained. Then, a separate list of husbands was prepared for each kebele, and computer-generated random numbers were used to select the required samples.

### Data collection tool

All the questionnaires were prepared in excel and changed online to extensible markup language (XML) form using files in Excel (Xls). Then, it is uploaded to the Open Data Kit (ODK) Aggregate, which is available in the Arba Minch University Health Demographic and Surveillance System (AMU-HDSS) office, and the form was downloaded to the data collection tablet/phone using ODK collect. The data collection tool was developed by reviewing different works of literature [1, 5, 25, 26, 27–29]. The tool consists of socio-demographic characteristics of the husband and their wives, obstetric characteristics, husband’s knowledge of pregnancy, and delivery husband’s knowledge of birth preparedness and complication readiness. Data concerning family economic status were collected by asking about ownership of selected assets common in the local area. Information regarding their wives was also gathered from the husband.

#### Data collection process

Twelve data collectors with experience in data collection using a tablet/phone and working for AM-HDSS were recruited. Three days (one day theoretical and two days practical) training about the study’s objectives and the procedure to be followed was given to data collectors and supervisors by the investigators and information communication technology (ICT) experts using a tablet before the actual data collection.

### Measurement

#### Husband’s knowledge of labor and childbirth danger signs

The participant was asked to mention any danger signs during labor, and delivery spontaneously. Knowledge of at least one obstetric danger sign during the period was coded as Yes or No [1].

#### Men’s involvement in BPCR

A husband of a woman was considered well prepared or involved if he made arrangements for at least three of the six components of BPCR practices (had identified a birth kit, had identified a skilled attendant, had saved money, knew where to go in case of an emergency, had prepared transportation in advance, or had contacted a blood donor in advance. Three out of six were chosen because previous studies have used 50% and above to determine who was well prepared [1, 28].

#### Skilled birth attendant

People with midwifery skills (midwives, doctors, and nurses with additional midwifery education) have been trained to be proficient in the skills necessary to manage normal deliveries and diagnose, manage or refer obstetric complications [30].

#### Health development army

It is a women-centered community organization that requires the establishment of health development teams of up to 30 households. Their main task is to identify and monitor pregnant women and focus on encouraging their members to use maternal health services [31].

#### Distance to the nearby health facility

Measured from the husband’s report on the walking hours to the health facilities. This was coded 1 if husbands reported the walking hours to reach the nearby health facility to be ≥ 30 min; otherwise, it was coded 0 [32].

### Data Processing and Analysis

Data were coded and entered through excel and exported to an open data kit (ODK) for data collection. The entered data was exported to STATA version 16 for analysis. Descriptive statistics such as frequency, mean, and standard deviation were computed to describe the variables of the study. The associations between BPCR practice and birth in the presence of a skilled birth attendant were assessed using multiple logistic regressions after adjusting for known confounders (Wives’ age, Wives’ education, husband’s education, Wives’ occupation, and wealth quintile). Explanatory variables on bivariate logistic regression analysis with *p <* 0.25 were entered into multivariable logistic regression analysis, and a p-value of less than 0.05 was used to declare statistical significance.

### Data quality control

A structured interviewer-administered questionnaire was prepared in English and translated into local languages, then translated back to English by an independent language expert to ensure its consistency. The translators were well-known translators for both languages. The instrument was pre-tested on 5% (72) of the sample size in Mirab Abaya district that was not included in the study and analysis. Amendments were made after the pretest. The training was given to data collectors and supervisors by the principal investigator. Investigators and supervisors checked on the spot and review all the questionnaires to ensure completeness and consistency of the information collected and immediate action was taken accordingly.

## Result

### Socio-demographic characteristics

All participants completed this study’s interview (response rate = 100%). Table 1 shows the socio-demographic characteristics of the respondents. The mean and ± SD age of the study participant was 33.7 ± 6.2 years, while that of their spouses was 29.1 ± 5.9 years. Regarding their educational status, more than one-third (462) of the husbands had formal education, whereas 29.3% of their spouses had formal education. The majority (87.8%) of the respondents are not involved in the health development army. Almost 70% of their spouse had ANC visits for their recent child, of which 43% had more than four visits. Three in four husbands did not know danger signs during childbirth.

**Table 1:** Background and reproductive characteristics of study participants (husband and Wives’) in Arbaminch zuria District, southern Ethiopia, 2021(N = 1432)

Background characteristics	N	%
**Husband’s age**		
18–25	122	8.52
26–35	745	52.03
36–40	393	27.44
>=41	172	12.01
**Wives’ age**		
16–24	308	21.51
25–29	460	32.12
30–34	365	25.49
>=35	299	20.88
**Husband’s education**		
Unable to read and write	656	45.81
Able to read and write	314	21.93
Formal education	462	32.26
Wives’ **education**		
Unable to read and write	742	51.82
Able to read and write	271	18.92
Formal education	419	29.26
**Husband’s occupation**		
Farmer	974	68.02
Daily Laborer	243	16.97
Merchant	75	5.23
Government employee	81	5.66
Other	58	4.05
**wives’ occupation**		
Unemployed	1,203	84.01
Employed	229	15.99
Wives’ **decision-making authority**		
Low authority	146	10.20
High authority	1,286	89.80
**Wealth index**		
The poorest	300	20.95
2nd quantile	287	20.04
Middle quantile	285	19.90
4th quantile	284	19.83
The richest	276	19.27
**Involvement in health development army**		
Yes	175	12.22
No	1,257	87.78
**ANC visit (for the youngest child)**		
Yes	995	69.53
No	312	21.80
I don’t know	124	8.67
**Frequency of ANC visit (for the youngest child) (N = 995)**		
Less than four times	469	47.14
Four and more times	431	43.32
Don’t know	95	9.55
**Complications during a previous pregnancy**		
Yes	238	16.62
No	1,194	83.38
**Knowledge about danger signs during labor and delivery**		
Had no knowledge	1,024	71.5
Had knowledge	408	28.5

### BPCR among husbands

As regards birth preparedness and complication readiness, the most common preparation was to save money (38.3%), then identified birth kit (17%), followed by identification of means of transportation (10.9%) and identification of which health facility to visit in case of emergency (10.1%) Few mentioned identification of blood donor. 14% of the respondents had not done any preparations before childbirth. One hundred forty men, 10.3% had made at least three preparations according to the concept of BPCR (Table 2).

**Table 2:** Components of birth preparedness and complication readiness practices among men participants in Arbaminch Zuria District, southern Ethiopia, 2021(N = 1432)

BP/CR	N	%
Saved money	549	38.34
Identified birth kit	244	17.04
Identified transport	157	10.96
Identified where to go for emergency	145	10.13
Identified skilled attendant	123	8.59
Identified blood donor	15	1.05
Made at least three steps	145	10.13

Multiple responses were possible

### The proportion of skilled birth attendant

In the current study, the prevalence of skilled birth attendants is 798 (55.8%) (95% CI: 53.2–58.3).

### The association between BPCR among husbands and wives use of institutional delivery

Husband involvement in BPCR, wives’ age, complications during a previous pregnancy, knowledge about danger signs during labour and delivery, and distance from facility associated with a skilled birth attendant in the binary logistic regression. However, in our study, there was no association between having 1–2 children and wives aged 30–34 years with a skilled birth attendant. After controlling for confounders through multivariable logistic regression, husbands who were involved in BPCR were more likely to have their spouse have a skilled birth attendants (AOR = 4.17, 95% CI: 2.50–6.92). Husbands whose wives had complications during previous pregnancy were 33% less likely to have a skilled birth attendant (AOR = 0.67, 95% CI: 0.49–0.92). Moreover, husbands whose houses were proximal to health facilities were predicted to using skilled birth attendants (3.93, 95% CI: 2.57–6.02) (Table 3).

**Table 3:** Association between selected socio-demographic characteristics, obstetric characteristics, birth preparedness, complication readiness, and skilled birth attendant

Characteristics	Facility delivery		Bivariate analysis Unadjusted OR OR (95%CI), p-value	Multivariate analysis Adjusted OR OR (95%CI), p-value
	Yes	No		
Husband involvement in BPCR*				
Involved	119	26	4.08 (2.63–6.33), ≤ 0.001	**4.17 (2.5–6.9), ≤ 0.001**
Non -Involved	679	606	1	1
**Wives’age**				
16–24	216	92	4.3(3.07–6.07), ≤ 0.001	2.4 (1.53–3.89), **≤ 0.001**
25–29	301	158	3.5 (2.58–4.75), ≤ 0.001	2.4 (1.66–3.55), **≤ 0.001**
30–34	176	189	1.7 (1.25–2.34), 0.001	1.5(0.95–1.82), 0.088
>=35	105	193	1	1
**Complications during a previous pregnancy**				
Yes	105	133	0.5 (0.43–0.75), ≤ 0.001	**0.67 (0.49–0.92), 0.012**
No	693	499	1	1
**Knowledge about danger signs during labor and delivery**				
Yes	262	145	1.6 (1.29–2.08), ≤ 0.001	**0.85(0.64–1.13), 0.280**
No	536	487	1	1
**Distance from a health facility (reachable in 30 min)**				
Yes	136	662	3.98 (2.65–5.97), ≤ 0.001	**3.93 (2.57–6.02), ≤ 0.001**
No	601	31	1	1
**Number of children**				
1–2	420	187	3.65 (2.78–4.8), ≤ 0.001	2.05(1.4–3.02), **≤ 0.001**
3–4	238	217	1.78 (1.35–2.36), ≤ 0.001	1.30(0.95–1.82), 0.088
>=5	140	228	1	1
**Occupation of the wives’**				
Employed	90	708	0.45(0.34–0.60), ≤ 0.001	0.39(0.28–0.54), **≤ 0.001**
Non employed	138	494	1	1

## Discussion

This paper highlights findings on BPCR among husbands and their association with skilled birth attendance in Southern Ethiopia. Husband involvement has been established to positively impact maternal and infant morbidity and mortality [33]. This study showed an association between husband involvement and skilled birth attendance, such that husbands involved in BPCR were more likely to have their spouses attend skilled birth. Findings were similar to an intervention study conducted in Uganda which showed a significant rise in the number of pregnant women delivering in health facilities due to community and facility-led interventions to strengthen husband involvement in skilled delivery [34]. In Zambia, a cross-sectional study established that women whom their spouses accompanied during ANC were more likely to deliver in a health facility and had partners’ support during postnatal care compared to those who were unaccompanied by spouses [33]. Another study conducted among mothers admitted to a hospital for emergencies in antenatal, labour, and postpartum in Rural Uganda showed that 42.9% and 43.4% were accompanied by their husbands to antenatal clinics and labor ward, respectively [35]. A systematic review and meta-analysis indicated that husband involvement was significantly associated with skilled birth attendance and postnatal care [36], leading to better-improved utilization of maternal health services. However, the same study concluded that husband involvement has more benefits during pregnancy and postpartum than during delivery [36]. From the studies cited, it is evident that husband involvement is associated with skilled deliveries, and more benefits are accrued when involvement begins right from antenatal care and not just at the point of delivery. The studies also indicate that community and facility-led interventions to strengthen husband involvement have increased uptake of skilled deliveries.

The husband’s involvement in BPCR, especially within communities in Sub-Saharan Africa, plays a vital role in the ability of women to prepare for birth and respond to obstetric complications [37]. Regarding ways men were involved in BPCR, findings were consistent with other studies conducted in Rwanda [38], Northern Nigeria [39, 40], and Nepal [41]. It is evident that very few men are involved in identifying skilled birth attendants and identifying potential blood donors but more involved in financial-related roles like saving funds and providing transport [37]. It is prudent to note that in Sub-Saharan Africa, including Ethiopia, most communities are patriarchal, and most men tend to regard childbirth and pregnancy as women roles. This seems to influence the extent to which they participate in BPCR [42]. Societies perceive men as providers; hence, in the case of BPCR, they will tend to perform financially related roles compared to other non-monetary related roles [7]. Therefore, programs targeting husband involvement in BPCR need to address aspects of socio-cultural contexts and their influence on gender roles that could be perceived as barriers. In the context of BPCR, men must be sensitized to the benefits of providing other support mechanisms, such as identification of skilled birth attendance and donors that will influence better delivery outcomes.

Moreover, educating men on danger signs during labor and delivery has significantly improved delivery outcomes [41]. This study established low levels of knowledge on danger signs of labor and delivery, which correlates with systematic review findings where men’s knowledge of pregnancy complications and level of maternal health utilization is low; an indication of decision making from an uninformed perspective [37]. Therefore, there is a need to design and execute programs that involve husband sensitization on danger signs during the perinatal period to improve their knowledge of emergency obstetric conditions and danger signs [43].

Distance from the facility was established as a factor for a skilled birth attendant in this study. The findings corroborate with results from a study conducted in Ethiopia, which established a higher proportion of skilled birth attendants among those whose facilities were closer to their residence [25, 26]. In Uganda, similar findings were observed where husband involvement was low in facilities more than 5 km from the residence [44]. It is important to note that the more accessible the facility is, the utilization of maternal health services. Therefore, integrating health system components that could influence access to healthcare facilities will contribute significantly to an improved skilled birth attendant. This could include interventions such as encouraging utilization of maternal shelters and support to access facilities, among others.

In the current study, husbands whose wives had complications during previous pregnancies were less likely to use skilled birth attendants than those without complications. The finding of our study is supported by Kea et al. [45]. This could be due to negative experiences with health facilities and issues regarding trust with health professionals. Therefore, communication and educational programs should emphasize attitudinal and behavioral changes while comforting parents with previous experiences.

## Conclusion and recommendations

This study shows that husband involvement in BPCR is significantly associated with a skilled birth attendant in Ethiopia. Therefore, it is imperative that when designing husband involvement programs targeting BPCR, clear strategies describing why men should be involved, what men need to do, how they need to be involved, and the extent of involvement based on differences in social and cultural contexts within various areas in Ethiopia should be integrated into programs to increase uptake of husband involvement in BPCR and thereby enhance skilled birth attendant.

## Electronic supplementary material

Below is the link to the electronic supplementary material.

Supplementary Material 1

Supplementary Material 2

Supplementary Material 3

## Data Availability

All data generated or analyzed during this study are included in this article [and its supplementary information files].
