# Supplementary material for: Birth preparedness and complication readiness among husbands and its association with skilled birth attendance in southern Ethiopia
Source: BMC Pregnancy Childbirth. 2022 Nov 19;22:852. doi: 10.1186/s12884-022-05147-3 (PMC9675132; doi:10.1186/s12884-022-05147-3)
Supplement: Supplementary file 1 — Supplementary Material 1 [file 12884_2022_5147_MOESM1_ESM.doc]

# ANNEXES

## Annex I: English Version Participant Information Sheet and Informed Voluntary Consent

## Annex II: English Version Questionnaire

| **Part 1: Socio -demographic characteristics** | | | |
| --- | --- | --- | --- |
| **S No** | **Question** | **Response** | **Skip** |
|  | Age | In completed years __________ |  |
|  | Your wife’s age | In completed years __________ |  |
|  | What is your religion? | 1. Protestant 2. Orthodox 3. Muslim 4. Other(specify)________ |  |
| 1. 1 | What is your current marital status? | 1. Married 2. Married but living apart |  |
|  | Is your marriage arranged or love marriage? | 1. Arranged marriage 2. Love marriage |  |
|  | Are you currently living with your partner? | 1. Yes 2. No |  |
| 1. 1 | How long have you been living with your partner? | ______________ |  |
| 1. 1 | What is the highest grade you have completed? | 1. Unable to read and write 2. Able to read and write 3. Primary school 4. Secondary school 5. College and above |  |
|  | What is the highest partners grade completed | 1. Unable to read and write 2. Able to read and write 3. Primary school 4. Secondary school 5. College and above |  |
|  | What is your occupation? | 1. Farmer 2. Daily Laborer 3. Trader/Merchant 4. Government employee 5. Non-governmental employee 6. Others (specify)_______ |  |
|  | What is your Partners occupation? | 1. Farmer 2. House wife 3. Merchant 4. Government employee 5. Non-governmental employee 6. Others (specify)_______ |  |
|  | Residency | 1. Urban 2. Rural |  |
|  | Are you member of health development army? | 1. Yes 2. No |  |
|  | What other membership in your community are you a member of? | _________ |  |
|  | Which media source do you use for health related information?  (Multiple answers are possible) | 1. Ekub 2. Radio 3. Community discussion 4. Newspaper 5. I don’t use any 6. Other (specify)_________ |  |
|  | In average how long does it take for you to reach to health facilities? | In minute _______ |  |
|  | Which health facility is nearest to you? | 1. Hospital 2. Health center 3. Health post |  |
| **Part II: Wealth index questions** | | | |
|  | **Asset type** | **Response** | |
|  | **Domestic animals** |  |  |
|  | Ox | No (0) | Yes (1) |
|  | Cow | No (0) | Yes (1) |
|  | Calf | No (0) | Yes (1) |
|  | Sheep | No (0) | Yes (1) |
|  | Goat | No (0) | Yes (1) |
|  | Horse | No (0) | Yes (1) |
|  | Donkey | No (0) | Yes (1) |
|  | Mule | No (0) | Yes (1) |
|  | Cock and Hen | No (0) | Yes (1) |
|  | **Durable assets** |  |  |
|  | Television | No (0) | Yes (1) |
|  | Radio | No (0) | Yes (1) |
|  | Electricity | No (0) | Yes (1) |
|  | Refrigerator | No (0) | Yes (1) |
|  | Conventional telephone | No (0) | Yes (1) |
|  | Mobile phone | No (0) | Yes (1) |
|  | Car | No (0) | Yes (1) |
|  | Motorcycle | No (0) | Yes (1) |
|  | Cycle | No (0) | Yes (1) |
|  | Cart | No (0) | Yes (1) |
|  | Gold, money | No (0) | Yes (1) |
|  | Ownership of owned living house | No (0) | Yes (1) |
|  | Ownership of agricultural land | No (0) | Yes (1) |
|  | **Productive assets** |  |  |
|  | Plough plow | No (0) | Yes (1) |
|  | Axe | No (0) | Yes (1) |
|  | Hoe | No (0) | Yes (1) |
|  | Shovel | No (0) | Yes (1) |
|  | Sickle | No (0) | Yes (1) |
|  | Modern beehive | No (0) | Yes (1) |
|  | Traditional beehive | No (0) | Yes (1) |
|  | **Housing characteristics** |  |  |
|  | Indoor plumping/ pipe water | No (0) | Yes (1) |
|  | Type of flooring | Earth/dung (0) | Cement/raw wood (1) |
|  | **Other household materials** |  |  |
|  | Bed | No (0) | Yes (1) |
|  | Table | No (0) | Yes (1) |
|  | Chair | No (0) | Yes (1) |
|  | Stove | No (0) | Yes (1) |
| **Part III: Reproductive History of the respondent** | | | |
|  | In your opinion which age is the right time for a girl to get married? | __________ |  |
|  | In your opinion which age is the right time for a girl to get pregnant? | __________ |  |
|  | At what age did you have your first child? |  |  |
|  | How many children ever born to you? |  | **If 1 go to 129** |
|  | How many of them are alive? |  |  |
|  | How old is your recent child? | __________ |  |
|  | What is the age difference between your last two children? | __________ |  |
|  | Between two consecutive children, how many years of intervals do you think is good? | __________year/s |  |
|  | How many children do you want to have? | ________________፟ |  |
|  | Have your wife ever experienced pregnancy terminated with abortion? | A. Yes  B. No  C. I do not remember  D. Unwilling to respond |  |
|  | If your answer to Q130 is “Yes”, how many times? |  |  |
|  | If ‘Yes’ to Q130, What were the means of abortion | 1. Spontaneous 2. Abortion because we don’ t want it 3. I don’t know 4. Others(specify) |  |
|  | Is your wife pregnant currently? | 1. Yes  2. No  3. I do not know | **135** |
|  | If the response to **Q134** is “Yes”, Is the pregnancy planed (wanted)? | 1. Yes 2. No |  |
|  | If the response to **Q134** is “Yes”, what is the duration of pregnancy (in months)? |  |  |
|  | If the response to **Q134** is “Yes”, have your wife ever been to ANC clinic for her current pregnancy? | - 1. Yes(write how many times)_____   2. No   3. I do not know |  |
|  | How many person live in your house  (Family size)? | ___________ |  |
|  | Do you have children from other  Partner/wife? | 1. Yes 2. No 3. Unwilling to respond |  |
|  | If your answer to is **Q138** “Yes” , how  many children do you have from other  Partner/wife? | _________ |  |
|  | Do you have more than one wife | 1. Yes 2. No | **142** |
|  | If yes you have more than one wife, how many wives you have? | ___________ |  |
| **Part VI: husband’s knowledge of danger signs during pregnancy, labor and postpartum.** | | | |
|  | If yes to Q 210, what are the danger signs you know? **Circle what respondents give, then probe (what else?) don’t read the options** | 1. Bleeding 2. Severe headache 3. Blurred vision 4. Convulsions 5. Swollen hands/face 6. High fever 7. Loss of consciousness 8. Difficulty breathing 9. Severe weakness 10. Severe abdominal pain 11. Accelerated/ reduced fetal movement 12. Water breaks without labor 13. Don’t know 14. Other (specify) |  |
|  | Could a woman die from this problem any of these problems? **(Q212)** | 1. Yes 2. No 3. Don’t know |  |
|  | Do you know about danger signs during labour and childbirth? | 1. Yes 2. No |  |
|  | If yes to **Q214,** what are danger signs during labour and childbirth?  **(Circle what respondents give, then probe (what else?) don’t read the options** | 1. Severe bleeding 2. Severe headache 3. Convulsions 4. High fever 5. Loss of consciousness 6. Labor lasting >12 hours 7. placenta not delivered 30 minutes after baby 8. Don’t know 9. Other (specify) |  |
|  | In your opinion, could a woman die from any of these problems? (**Q215**) | 1. Yes 2. No. 3. Don’t know |  |
|  | Do you know about danger signs during postnatal period? | 1. Yes 2. No |  |
|  | If yes, what are the danger signs during postnatal period ? **(Q217) Circle what respondents give, then probe (what else?) don’t read the options** | 1. Severe bleeding 2. Severe headache 3. Blurred vision 4. Convulsions. 5. Swollen hands/face 6. Malodorous vaginal discharge 7. Loss of consciousness 8. Difficulty breathing 9. Severe weakness 10. Don’t know 11. Other (specify) none |  |
|  | Could a woman die from any of these problems **(Q219**)? | 1. Yes 2. No 3. I don’t know |  |
| **Part III: Husbands involvement in maternal and child care regarding their spouse most recent pregnancy**  **3.1 Husbands involvement during antenatal period** | | | |
|  | Did your spouse receive antenatal checkups? | 1. Yes 2. No 3. I don’t know |  |
|  | Do you think antenatal checkups are necessary? | 1. Yes 2. No |  |
|  | If yes to Q302 , how many times did she receive | 1. Less than 4 times 2. 4 and more times 3. Don’t know |  |
|  | At what gestational age did she start first visit? | _______ |  |
|  | Did you accompany your wife for ANC? | 1. Yes 2. No |  |
|  | If yes, how many times did you accompany her? | ____________ |  |
|  | Did you physically entered the ANC room together with your partner | 1. Yes 2. No |  |
|  | Do you have experience of discussion with health care provider about your wife’s last pregnancy and childbirth? | 1. Yes 2. No |  |
|  | Did you discuss with your wife regarding the choice of health care provider for the last birth? | 1. Yes 2. No |  |
|  | If yes, which type of provider did you recommend? | 1. Specialist 2. General physician 3. Nurse/midwife 4. HEW 5. TBA |  |
|  | Were you counseled and tested for HIV during partner’s pregnancy. | 1. Yes 2. No |  |
|  | Did you request your partner to be tested for HIV during pregnancy | 1. Yes 2. No |  |
|  | Did you took time to find out what went on during partner’s ANC visits | 1. Yes 2. No |  |
|  | Did you remind your partner’s ANC follow-up schedule | 1. Yes 2. No |  |
|  | Did you attend the health information with your wife at health facility? | 1. Yes 2. No |  |
| **3. 2 Birth preparedness and complication readiness (BP/CR) of men in previous pregnancy** | | | |
|  | Do you know the components of birth preparedness and complication readiness | 1. Yes 2. No |  |
|  | If yes to the above question, what are the components of birth preparedness and complication readiness ?  **(Circle what respondents give, then probe (what else?) DON’T read the options** | 1. Arranging for postpartum cultural food expenses 2. Saving money for Mother’s health care 3. Identifying a mode of transportation 4. Identifying place of delivery 5. Clean clothes & other materials for Baby/Mother’s 6. Savings for emergencies Awareness on emergency & it immediately action 7. Arrangement for skilled birth assistance 8. Identifying decision maker for emergency 9. Arranging blood donors Identifying date of birth |  |
|  | Where did your spouse deliver your last baby? | 1. Home 2. Public health institution 3. Private facility |  |
|  | Did you accompany your wife to the place where she gave birth? | 1. Yes 2. No |  |
|  | If yes to **Q 324** did you enter and attend your wives labour and delivery in the room? | 1. Yes 2. No |  |
|  | Who assisted the delivery? | 1. Doctor 2. Nurse 3. Traditional birth attendant 4. Health extension worker 5. Relative/friend |  |
|  | Who made the final decision where to give birth? | 1. Yourself 2. Your spouse 3. Jointly with your spouse 4. Other family member 5. Health professionals 6. Friend 7. Other (specific ) |  |
|  | How was the child born | 1. Normal spontaneous vaginal delivery 2. Assisted delivery (forceps or vacuum 3. Cesarean section |  |
|  | During labour and birth did your wife experience any serious problem related to birth? | 1. Yes 2. No |  |
|  | If yes to **Q325** what problems did she experience during child birth?  (tick as the participant mentions) | 1. Severe bleeding 2. Severe headache 3. Blurred vision 4. Convulsions. 5. High fever 6. Loss of consciousness 7. Labour lasting more than 12hours 8. Placenta not delivered after 30minutes of 9. Other (specific) |  |
